# Resurrecting social infrastructure as a determinant of urban tuberculosis control in Delhi, India

**DOI:** 10.1186/1478-4505-12-3

**Published:** 2014-01-17

**Authors:** Shivani Chandra, Nandini Sharma, Kulanand Joshi, Nishi Aggarwal, Anjur Tupil Kannan

**Affiliations:** 1 Office of the World Health Organization (WHO) Representative to India, WHO Country Office, New Delhi 110011, India; 2 Department of Community Medicine, Maulana Azad Medical College, Government of National Capital Territory of Delhi, New Delhi 110002, India; 3 Office of Chief Minister Delhi, Government of National Capital Territory of Delhi, New Delhi 110002, India; 4 Department of Biostatistics, New Delhi TB Centre, New Delhi 110002, India; 5 Department of Community Medicine, University College of Medical Sciences, Government of National Capital Territory of Delhi, New Delhi 110095, India

**Keywords:** Decline in new TB cases, Social capital, Social determinants, Social infrastructure, Tuberculosis, Universal coverage, Urban TB

## Abstract

**Background:**

The key to universal coverage in tuberculosis (TB) management lies in community participation and empowerment of the population. Social infrastructure development generates social capital and addresses the crucial social determinants of TB, thereby improving program performance. Recently, there has been renewed interest in the concept of social infrastructure development for TB control in developing countries. This study aims to revive this concept and highlight the fact that documentation on ways to operationalize urban TB control is required from a holistic development perspective. Further, it explains how development of social infrastructure impacts health and development outcomes, especially with respect to TB in urban settings.

**Methods:**

A wide range of published Government records pertaining to social development parameters and TB program surveillance, between 2001 and 2011 in Delhi, were studied. Social infrastructure development parameters like human development index along with other indicators reflecting patient profile and habitation in urban settings were selected as social determinants of TB. These include adult literacy rates, per capita income, net migration rates, percentage growth in slum population, and percentage of urban population living in one-room dwelling units. The impact of the Revised National Tuberculosis Control Program on TB incidence was assessed as an annual decline in new TB cases notified under the program. Univariate linear regression was employed to examine the interrelationship between social development parameters and TB program outcomes.

**Results:**

The decade saw a significant growth in most of the social development parameters in the State. TB program performance showed 46% increment in lives saved among all types of TB cases per 100,000 population. The 7% reduction in new TB case notifications from the year 2001 to 2011, translates to a logarithmic decline of 5.4 new TB cases per 100,000 population. Except per capita income, literacy, and net migration rates, other social determinants showed significant correlation with decline in new TB cases per 100,000 population.

**Conclusions:**

Social infrastructure development leads to social capital generation which engenders positive growth in TB program outcomes. Strategies which promote social infrastructure development should find adequate weightage in the overall policy framework for urban TB control in developing countries.

## Background

Over the past decade, there have been important advances in the global fight against tuberculosis (TB) and towards achievement of the Millennium Development Goals. The global TB response has become more equitable by placing patients at the centre of treatment, prevention, and care. In the year 2006, the World Health Organization (WHO) and the Stop TB Partnership articulated the impact targets for TB cases and deaths in context with the United Nations Millennium Development Goals [1]. With the annual rate of decline in incident TB cases at 1.3% globally and 2.2% in the South East Asia region [2], the 2015 Millennium Development Goal targets are achievable, but TB elimination by 2050 remains a distant dream [3].

TB has traditionally been considered as an archetypal disease of poverty which tends to involve a disproportionately large number of underprivileged members of society. Therefore, in order to accelerate economic and social growth and consequently reduce the global burden of TB, it is essential to fight TB and poverty together. The inequities in health in terms of affordable access to quality care services and the avoidable health inequalities in terms of standardised care practices arise because of the circumstances in which people live and grow. As TB is a medical condition with significant social dimensions, it is essential therefore, that while addressing health equity, any systematic framework for assessment of health care must look beyond medical excellence as good health means not only to reduce suffering but to expand a person’s life in order to be able to do what s/he wishes to achieve.

In order to accelerate the annual rate of decline in TB incidence required for TB elimination, concomitant efforts need to be made towards implementation of pro-poor, pro-health policies addressing health inequalities. Ambit of health care services which encompass socio-economic arrangements is vital for the universal health coverage mandate which preludes the achievement of the Millennium Development Goals [4].

### Caring for urban TB

The United Nations estimates that the world’s urban population will grow by 2 billion before 2030 [5], with a large part of this growth focusing in the unplanned urban slums. Continuous inflow of migrants and mushrooming of unauthorized colonies in slum dwellings have increased the vulnerability of health risks among the urban poor. This, coupled with the fact that TB disease has medical and social implications, highlight the role of social development in improving program performance in an urban area.

### Delhi demographic profile

In India, urbanization is fast becoming the defining process in shaping the course of social transformation, though the urban advantage evades the urban poor, which form one fourth of India’s population. Delhi, the capital city of India, is one of the largest urban agglomerations with more than 16 million population (Table 1). The phenomenal population growth in Delhi is predominantly due to large scale migration in the State as a consequence of which half of Delhi’s population lives in slums and other urban poor habitation. Besides this influx, around two million commuters visit Delhi every day, primarily for education and employment. The unprecedented growth in the slum population indirectly reflects on the poverty, substandard living conditions, and marginalization from basic health services [6]. This has put tremendous pressure on urban infrastructure in the State to provide access to basic public amenities to its residents. As health demands not only health care but also other factors such as economic and social arrangements and the fact that the health system alone does not possess the tools to solve all its health challenges, the Government of Delhi took a call for an epistemic approach to healthcare thus defining the need for good governance initiatives which promote the development of social infrastructure.

**Table 1:** Demographic profile and TB program performance of Delhi for the years 2001 and 2011

**Delhi demographic profile**^**a,b**^	**Year 2001**	**Year 2011**
Delhi population	13,850,507	16,753,235
Decadal growth rate	47.0%	21.0%
Net migration rate	12.7%	17.5%
Population density (per square km)	9,340	11,297
Sex ratio (Females per 1,000 males)	821	866
Literacy rates (%)	82	86
Per capita income (INR)	38,864	148,608
**Urban Delhi profile**^**c**^	**Year 2001**	**Year 2011**
Population living in urban areas	93.0%	97.5%
Population growth rate in urban areas	51.3%	26.6%
Population living in one-room dwelling units	38.1%	32.2%
Average number of household members	4.9	5.2
Slum population residing in urban areas	15.7%	19.6%
Unauthorized settlements (shanty clusters/unauthorized/resettlement colonies)	67.5%	46.0%
**TB program performance in Delhi**^**d**^	**Year 2001**	**Year 2011**
Percentage of State Government budget on health^c^	7	12
Number of doctors in government hospitals per 10,000 population^c^	1.9	3.8
Number of treatment centres (DOT centres)	51	585
Number of private sector engagements	10	231
Number of TB suspects examined	153220*	164392
Number of new TB case notification rates per 100,000 population	229	214
TB death rates (%)	3.1	2.2
Number of lives saved (all types of TB patients)	4775	9076
Lives saved (all types of TB patients) per 100,000 population	35	51

Data Source: ^a^Office of the Registrar General and Census Commissioner, Ministry of Home Affairs, Government of India. ^b^Office of the Registrar General and Census Commissioner, Ministry of Home Affairs, Government of India. ^c^Urban Health Division, Ministry of Health and Family Welfare, Government of India. ^d^Revised National Tuberculosis Control Program Delhi, Government of National Capital Territory of Delhi (http://www.dotsdelhi.org/program-performance.php) *Data available from the year 2005 onwards in Annual TB Report, 2006 at the website of Revised National Tuberculosis Control Program, Government of India (http://www.tbcindia.nic.in/pdfs/Annual%20Report%20TB%202006.pdf).

### Social infrastructure development in Delhi

Social infrastructure denotes the services and processes which enhance community capacity [7]; it includes development in health (individual and family health), education, information, housing, employment, art and culture, transport and public safety. There has been a substantial increase in social development parameters, such as growth in health and education infrastructure, in Delhi since the year 2000. Several initiatives of the State Government are geared towards creating effective partnerships with community groups for the development and management of the social infrastructure. One of the major initiatives towards building the community capacity in Delhi is the ‘Bhagidari’ (Partnership) program rolled out by the Government of Delhi in the year 2000 with Resident Welfare Associations (Neighbourhood Communities) for the improvement of education, health, and civic amenities in their locality.

**Figure 1:**
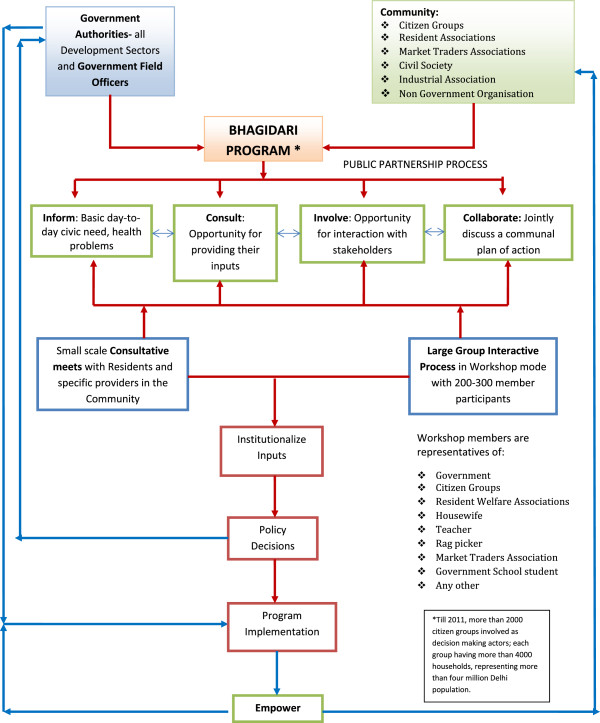
**Bhagidari program: government-citizen partnership with public authorities, private agencies, and public as end-users.** *Courtesy: Office of Chief Minister Delhi, Government of National Capital Territory of Delhi.

The Bhagidari program is a participatory governance initiative which promotes Government–Community–Citizen engagement under its framework to make the systems more responsive to its citizens [8,9]. The Bhagidars, or partners, represent local residents and vulnerable people. The involvement of citizens in the policy framework has led to development of sense of ownership by the citizens, a shift of mind-sets from that of ‘Government as Provider’ to ‘people as empowered’. It has taken the initiative of ‘partnering in governance’ with a progressive work-culture of ‘let’s work together’ and has produced new collective actors of local associations and popular social groups. In this initiative, residents act as active partners in decision making, they discuss issues affecting effective delivery of civic services with government representatives, and propose a local plan of action pertaining to the desired civic need. In addition to participatory governance, during the small-scale consultative meets and large-scale interactive forums, Bhagidars are informed about the several initiatives rolled out by the State Government for the social security and welfare of residents. With the community getting actively involved through Bhagidari programs, other social sector initiatives by the State Government, such as the ‘Mission Convergence’ scheme^a^ for collaboration with civil society and various government departments, illness assistance schemes like the ‘Delhi Arogya Nidhi Scheme’^b^, several demand-based interventions like the ‘Ladli’ scheme^c^ for incentivizing mandatory education of the girl child, ‘Rashtra Swasthya Bima Yojana’^d^ which is a national health insurance scheme that reduces out-of-pocket expenditure for health care and lessens considerable financial burden on the poorest of the poor, and ‘Janani Suraksha Yojana’^e^ which is a conditional cash transfer scheme that incentivizes women to give birth in health facilities, have all received enhanced advocacy and outreach among the beneficiaries. This has also helped a substantial number of TB patients to get benefits from these social protection schemes [10]. The Bhagidari program is the process for social infrastructure development; the program was extended to all slums of Delhi in the year 2007 through the ‘Sanjha Prayas’^f^ initiative under the Bhagidari program and through collaboration with existing social welfare schemes. By 2011, approximately 2,000 citizen groups were involved as decision-making actors, representing more than four million of Delhi’s population. The framework in Figure 1 explains the mechanism adopted for public participation in the Bhagidari program.

### Social capital generation from social infrastructure development

The main essence of Social Capital is that it refers to the trust, civic norms, and networks that enable collective action and improve market performance by reducing transaction costs [11,12]. It is the fundamental requirement for any health equity intervention package intending to address wider public health needs. Thus, it’s inclusion in a social sector program yields clear benefits [13]. Social infrastructure includes a wide range of activities and facilities which support the formation of social capital; building social infrastructure is integral to the development of sustainable communities [14]. The participatory governance concept as introduced through the Bhagidari program leads to group participation, building trust and confidence, self-reliance, and income generation in the community. This causes an improved standard of living leading to social infrastructure development and growth in social determinants, which further strengthens social capital. Social capital generated through social infrastructure development provides a platform for community intervention to resolve issues related to health and civic amenities in the locality, thus building stronger communities.

The study supports social infrastructure development underscoring utilization, access, equity, and community empowerment for urban TB control in developing countries. The present paper attempts to study the interrelationship between social infrastructure development and TB program impact in Delhi. Social infrastructure development parameters, such as the human development index (HDI) along with other indicators reflecting patient profile and habitation in urban settings, were selected as social determinants of TB. These include adult literacy rates, per capita income, net migration rates, percentage growth in slum population, and percentage of urban population living in one-room dwelling units. The study was conducted with an objective to understand the correlation between decline in new TB cases and social sector growth in the State of Delhi.

## Methods

Data was compiled from program surveillance records of new TB patients registered each year under the Revised National TB Control Program, Delhi. Published records of the Government of National Capital Territory of Delhi and the Government of India were used to study the selected social determinants of TB between the years 2001 and 2011 in Delhi.

### TB impact indicator

The TB impact indicator used in the study is the annual rate of decline in new TB cases per 100,000 population [15]. Measurement of new TB cases is based on the WHO policy package for calculating rates of TB incidence, which states that in countries where TB programs have focused on a systematic approach for assessing the quality and coverage of TB surveillance data, then the data from program records is ‘certified’ as a direct measure of TB incidence and is a close proxy for TB incidence in the area [16]. Consequently, the logarithmic rate of decline in TB over successive years of program implementation per 100,000 population was calculated based on the TB incidence values from country program surveillance data for the State of Delhi [17].

### Measure of social infrastructure development

Social infrastructure development was studied in context with social capital generation. Being a multifaceted concept, social capital cannot be symbolized by any single direct indicator [18]. Therefore, in the study, social determinants which pose as indirect indicators of social capital, such as HDI, along with indicators reflecting patient profile and habitation in slums, such as adult literacy rates (>7 years age), per capita income, net migration rates, percentage growth in slum population, and percentage of urban population living in one-room dwelling units have been chosen as a measure of social development. The indicators used for the analysis have been compiled from published database of the Government of India and the Government of National Capital Territory of Delhi [19-23]. The HDI for Delhi was 0.687 and 0.740 for the years 1996 and 2006, respectively, ranking it fourth in the country. The average annual national HDI growth rate (percentage) is 1.56 and the HDI growth rate for Delhi has been computed based on the national HDI projections. Though not included in the analysis, government effectiveness was also studied to understand the role of a stable government towards holistic sustainable development.

### Interrelationship between TB impact and social development

After controlling for the impact HIV on TB incidence, univariate linear regressions were carried out to illustrate the relationships between each independent and dependent variable. Independent variables chosen were HDI, adult literacy rates, per capita income, net migration rates, percentage growth in slum population, and percentage of urban population living in one-room dwelling units and the dependent variable analysed was new TB cases per 100,000 population. Significance in univariate correlation was defined as r^2^ >0.2 and *P* <0.05 two-tailed. SPSS Version 16 (Copyright SPSS Inc.) was used for the analysis.

## Results

### Impact of the TB program in Delhi

**Figure 2:**
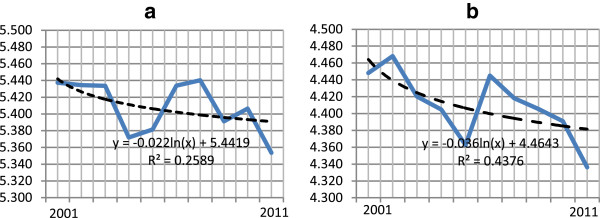
**Logarithmic decline in tuberculosis (TB) notification rate per 100,000 population; Delhi, 2001–2011. (a)** New TB patients; **(b)** New smear-positive TB patients.

Investigation of TB surveillance records identified 492,683 cases of all forms of TB registered in the Revised National TB Control Program Delhi between the years 2001 and 2011. There was an increment of 46% lives saved among all types of TB cases per 100,000 population between 2001 and 2011. In numbers, this amounts to 9,076 lives saved in 2011 as against 4,775 lives saved in 2001. In Delhi, declining trends were observed in new TB case notification rates during the study period. The number of new TB cases notified under the Revised National Tuberculosis Control Program steadily declined from 229/100,000 population in 2001 to 214/100,000 population in 2011, a decline of 7% over ten years since 2001. During the study period, the logarithmic decline in new TB (all forms of TB) patients was at the rate of 5.41 TB cases per 100,000 population in Delhi (Table 2, Figure 2a, b). The logarithmic decline in incidence of new smear positive (infectious) TB patients was found to be significantly higher as compared to the logarithmic rate of decline in all forms of new TB patients.

**Table 2:** Decline in new TB patients (all forms of TB) and new smear-positive TB patients per 100,000 population; Delhi, 2001–2011

**New TB patients (all forms of TB) per 100,000 population**^**c **^**registered under the Revised National TB Control Program in Delhi (2001–2011)**					**New smear positive TB patients per 100,000 population**^**c **^**registered under the Revised National TB Control Program in Delhi (2001–2011)**			
**Year**	**New TB patients**^**a**^	**Rate (per 100,000)**	**95% Confidence interval (±)**	**Log rate**	**New smear positive TB patients**^**b**^	**Rate (per 100,000)**	**95% Confidence interval (±)**	**Log rate**
2001–2002	31,718	229.84	2.53	5.437	11,794	85.46	1.54	4.448
2002–2003	31,856	229.18	2.54	5.435	12,119	87.19	1.56	4.468
2003–2004	34,121	229.00	2.62	5.434	12,384	83.11	1.58	4.420
2004–2005	33,155	215.29	2.59	5.372	12,604	81.84	1.60	4.405
2005–2006	34,778	217.36	2.65	5.382	12,554	78.46	1.59	4.363
2006–2007	36,873	229.03	2.73	5.434	13,717	85.20	1.66	4.445
2007–2008	38,261	230.49	2.78	5.440	13,768	82.94	1.67	4.418
2008–2009	37,532	219.49	2.75	5.391	14,002	81.88	1.68	4.405
2009–2010	39,222	222.85	2.81	5.407	14,207	80.72	1.69	4.391
2010–2011	37,655	213.99	2.76	5.354	13,245	75.26	1.66	4.336
			Average	5.408			Average	4.410
			Slope	-0.005			Slope	-0.009
			Standard deviation	0.003			Standard deviation	0.003
			95% Confidence interval (±)	0.014			95% Confidence interval (±)	0.015
			Pearson’s coefficient	-0.557			Pearson’s coefficient	-0.749
			*P* value	0.050*			*P* value	0.006**
			(one-tailed)				(one-tailed)	

Notes. *P <0.05 level, **P <0.01 level.

Data Source ^a,b^Program surveillance records, Revised National Tuberculosis Control Program, Government of National Capital Territory of Delhi and Central TB Division, Ministry of Health and Family Welfare, Government of India.

Data Source ^c^Census of India, Office of the Registrar General and Census Commissioner, Ministry of Home Affairs, Government of India.

### Interrelationship between TB impact and social development in Delhi

Correlation and univariate linear regressions were carried out to illustrate the interrelationship between decline in new TB cases and social determinants of TB. Tables 3 and 4 summarize the analysis results.

**Table 3:** Correlation matrix: endogenous and exogenous TB variables in Delhi from 2001–2011 (n = 10)

**Parameters**		**Human development index**	**Literacy rates**	**Per capita income**	**New TB cases per 100,000 population**	**Net migration rates**	**Percentage growth in slum population**	**Percentage of urban population living in one-room dwelling units**
**Human development index**	Pearson correlation	1	0.951**	0.981**	-0.846*	-0.340	0.998**	-0.998**
	Sig. (two-tailed)		0.004	0.001	0.03	0.51	0.001	0.001
**Literacy rates**	Pearson correlation	0.951**	1	0.934**	-0.784	-0.435	0.957**	-0.956**
	Sig. (two-tailed)	0.004		0.006	0.06	0.39	0.003	0.003
**Per capita income**	Pearson correlation	0.981**	0.934**	1	-0.774	-0.222	0.989**	-0.990**
	Sig. (two-tailed)	0.001	0.006		0.071	0.672	0.001	0.001
**New TB cases per 100,000 population**	Pearson correlation	-0.846*	-0.784	-0.774	1	0.538	-0.846*	0.844*
	Sig. (two-tailed)	0.03	0.06	0.071		0.27	0.03	0.03
**Net migration rates**	Pearson correlation	-0.340	-0.435	-0.222	0.538	1	-0.333	0.328
	Sig. (two-tailed)	0.51	0.39	0.672	0.27		0.52	0.53
**Percentage growth in slum population**	Pearson correlation	0 · 957**	0.957**	0.989**	-0.846*	-0.333	1	-0.999**
	Sig. (two-tailed)	0.003	0.003	0.001	0.03	0.52		1.122E-09
	
**Percentage of urban population living in one-room dwelling units**	Pearson correlation	-0.998**	-0.956**	-0.990**	0.844*	0.328	-0.999**	1
	Sig. (two-tailed)	0.001	0.003	0.001	0.03	0.53	1.122E-09	

*P <0.05 level, **P <0.01 level.

**Table 4:** Univariate linear regression: impact of social determinants on number of new TB cases per 100,000 population in Delhi during the years 2001–2011

**Social predictors**	**r**^**2**^	**F**	**Unstandardized coefficient**		**Standardized coefficient**	***t***	**Sig.**
			**B**	**Standard error**	**Beta**		
**Human development index**	0.716	10.08	-0.886	0.279	-0.846	-3.17	0.03*
**Literacy rates**	0.62	6.4	0.401	0.877	0.462	1.344	0.311
**Per capita income**	0.774	5.98	-0.139	0.057	-0.774	-2.447	0.071
**Net migration rates**	0.289	1.63	0.025	0.02	0.538	1.27	0.27
**Percentage growth in slum population**	0.716	10.08	-0.043	0.013	-0.846	-3.17	0.03*
**Percentage of urban population living in one-room dwelling units**	0.712	9.89	0.012	0.003	0.843	3.14	0.03*

Notes. *p<0·05, Dependent Variable: Number of New TB Cases per 100,000 population.

The social determinants which showed significant association with decline in new TB cases were HDI (negative association), percentage growth in slum population (negative association), and percentage of urban population living in one-room dwelling units (positive association). Adult literacy rates and per capita income showed a non-significant negative association with the decline in new TB cases. Net migration rate though positively associated, was not a significant predictor of decline in new TB cases during the study period.

HDI and percentage growth in slum population showed significant negative correlation with new TB cases per 100,000 population, indicating that an increase in HDI and percentage growth in slum population tends to cause a significant reduction in number of new TB cases. Percentage of urban population living in one-room dwelling units showed significant positive correlation to new TB cases, which suggests that residents of crowded dwellings have higher incidence of TB. There was no significant association with literacy rates and per capita income indicating that it is ostensibly more important to be aware than educated and that TB is not only a disease of the poor. Net migration rates, though positively correlated, were not significant, which suggests that migration has no effect on TB incidence in the State.

As detailed in Table 4, univariate regression showed that HDI and percentage growth in slum population had significant negative regression weights with the dependent variable. One-room dwelling units showed positive regression weights while literacy rates, per capita income, and net migration rates showed no significant contribution to the new TB cases in the State. The results of univariate linear regression suggest that a significant proportion of new TB cases could be predicted by HDI levels (negative association), percentage growth in slum population (negative association), and one-room dwelling units (positive association) in the State. Translating it into numbers, 0.887 new TB cases will decrease in the city with every one point increase in HDI (*P* <0.05). Additionally, the r^2^ values indicate that approximately 72% of the variation in new TB cases is predicted by HDI levels, which is a composite assessment of standard of living (health index, literacy index, and income index) in a community. Similar results were observed with percentage growth in slum population, indicating that a significant reduction in new TB cases is associated with one unit increase in slum population (*P* <0.05). A significant positive association was observed with percentage of population living in one-room dwelling units and new TB cases (*P* <0.05).

With regards to literacy rate, per capita income, and net migration rate variables, no significant associations could be detected to the dependent variable. The insignificant positive regression weights shown by literacy rates indicate that level of awareness is more essential than literacy status for availing TB care services. Per capita income showed non-significant negative regression weights, suggesting that rising income level has little or no impact on TB disease. Similarly, net migration rates showed no significant contribution to new TB cases in the community. However, the effect of migration on disease burden (which includes both new and previously treated cases) requires further study.

## Discussion

Through this study we get a clear message that there is an inherent synergy between social infrastructure development and TB program impact in Delhi. As shown, exogenous factors, such as social determinants of TB, supplement endogenous factors, such as TB program performance, thus complementing each other’s efforts towards TB control dynamics.

One of the consequences of rapid urbanization in the developing world is the continuous growth of slums. Many health outcomes are worse in slums than in neighbouring urban areas or even rural areas. Over the years, growth in cities has affected several socio-demographic and economic factors in urban communities. Poor housing conditions and overcrowding are synonymous with slum dwellings and have been implicated in the spread of TB [24]. Due to the inability to plan for adequate social infrastructure in urban areas, these problems have also aggravated. In Delhi’s scenario, it was observed that a rise in slum population had a significant effect on the decline of new TB patients. This suggests that the holistic social development achieved due to growth in social infrastructure under the Bhagidari initiative has been instrumental in resolving issues related to health and civic amenities in the slum areas of the city.

In addition, it has also been observed that people who live in the same house with a TB patient are at greatest risk of exposure to TB [25]. In an essay on slum health [26], the authors have suggested to harness the existing resident structure and social capital for provision of basic services in slum dwellings. One such initiative for involvement of residents in the neighbourhood is the Bhagidari approach, detailed in this study.

Another exogenous factor which affects TB incidence is migration. Several studies performed in countries like New Zealand [27], United States [28], and Singapore [29], have shown that the prime reason for TB incidence not decreasing in these countries is the migration of TB-infected people from high incidence countries, a finding which was not observed in the Delhi scenario. Sound prevention strategies which involve communities in the improvement of the health of migrants instead of focusing on their parent birth place have previously been suggested [30]; this fact gets restated herein. Migration has shown no effect on new TB cases in the city; this reaffirms the need to focus on delivering a holistic package of services to all residents through social capital initiatives which instil community participation.

In 2002, Singh et al. [31] suggested the need for extensive health education by community involvement to create awareness about TB in the slum communities of Delhi. Although literacy rates did not contribute to the correlation matrix in our study, the positive regression weights indicate that a level of awareness is more essential than literacy status for availing TB program services.

It is notable that the largest impact of any public health intervention is at the community level [32]. The WHO Commission on Social Determinants of Health advocates community development as one of the theme areas of intervention for ensuring equity in population health [33]. Researchers have suggested that inter-sectoral collaboration, along with community participation, is essential for achieving equity in program performance and health outcomes [34]. Their report emphasizes the fact that the final fight for equity rests with the public sector. Despite the predominance of the private health sector in urban cities, it has been observed that the health needs of the socio-economically disadvantaged section of the population are rarely met by them. Invariably, this burden is shouldered by the public health sector, the performance of which depends upon the development of social infrastructure. This implies that countries like India with a large public sector hub will have the maximum impact on population health through community development strategies. Building community capacity will ensure substantial progress in the Government’s effort to promote equity for all, as has been observed in the Delhi scenario.

The notion that addressing social determinants through community involvement corrects health inequities in a community is not just rhetoric but a reality, requiring workable ideas for action. Several authors [35,36] have posited the need for research on community-based interventions to understand the biological and social phenomena driving the TB epidemic. Partnerships which involve actors from within and beyond the health sector will facilitate a better understanding of the process of linking social determinants to TB. The community participation approach emphasized in this study reiterates the involvement of residents as third partners leading to improved health and development outcomes, especially with respect to TB.

Researchers have tried to explore the causal mechanism behind the positive relationship between social capital generation and TB program outcomes. In their study on correlation between social capital and TB, Holtgrave and Crosby [37] have suggested that social capital is highly predictive of TB program outcomes. Regression models have established the synergy between social determinants and TB impact indicators, especially TB incidence [38]. In countries like Bangladesh, Senegal, Thailand, and Zambia, the rate of decline in TB has been attributed to social sector reforms [39]. This reflects the need for several exogenous factors to work in tandem in order to affect the social and health outcomes in an area [40]; our study also corroborates these findings.

The study surmises that adoption of a meso-level public-private interface fosters collaboration between all principal actors (public authorities, private sector, and the public as an end-user) in the community. Researchers have ascertained that partnerships which use third party interface show a higher contribution to TB case detection [41]. Having seen the challenges faced by the TB program in urban areas with weak public health infrastructure and huge out-of-pocket expenditures, the TB program policy makers are exploring new strategies to leverage public-private partnerships with the help of public-private interface agencies for collaboration with various providers in urban settings. It is extremely important that while drafting such strategies, the public as end-users are engaged in the policy framework for improved acceptability and better monitoring of these services. Though extensive leverage for supply-side markets are in vogue globally, it is equally important to have a demand-side empowerment for further leveraging these initiatives without which there will be equity deficit in service delivery.

### Recommendations

Emerging from this discussion is the fact that a holistic approach addressing social determinants in an urban set-up is *sine qua non* for TB control. The significance of social infrastructure development as a positive catalyst for achieving broader public policy outcomes in urban TB control requires renewed attention and resurrection by researchers for its adoption in policy design. Thus, a strong policy inducement promoting social infrastructure development under a decentralized administrative initiative like the Bhagidari program needs to be acknowledged in the public health policy framework for urban TB control in the developing world.

**Figure 3:**
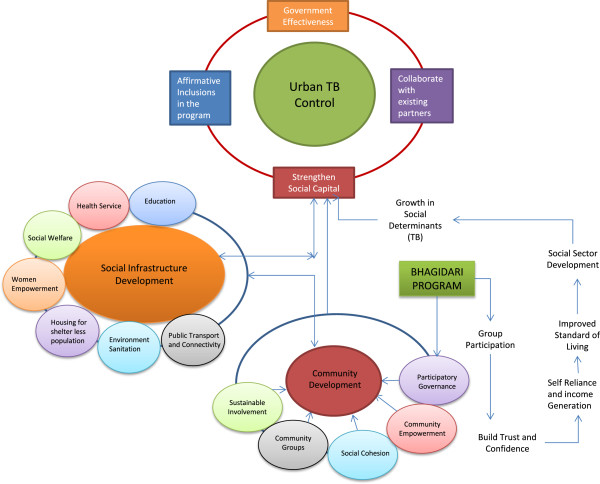
Schematic framework for urban tuberculosis control.

Planning and provision of social infrastructure needs coproduction and collaboration with various sectors. The study strongly echoes that caring for urban TB does not necessarily imply a separate system setup, but a need to develop an integrated model by collaborating with existing partners for sustainability. Researchers [42,43] have identified a set of parameters which could be incorporated in the TB program monitoring indicators at service delivery level. Based on the observations of this study, few systemic interventions are recommended for urban TB control in developing countries (Table 5). These interventions will not only enhance the program’s performance by harnessing the community’s potential but will also help in making the social opportunities more accessible to TB patients in addition to the availability of health services. A schematic framework depicting the synergy between social infrastructure development and urban TB control has been developed on the basis of the above recommendations (Figure 3).

**Table 5:** Shift in systemic intervention for urban TB control in developing countries

**Thrust areas**	**Work plan**
**Strengthen social capital**	• Adopt community intervention strategies which support development of social infrastructure
	• Create opportunities to encourage people’s participation in decision-making and community activities
	• Collaborate with elected representatives and community self-help groups for the public health responsibility of their community
**Collaborate with existing service providers**	• Liaison with the Ministry of Urban Development for Urban Self Employment program, Urban Women Self Help programs. Availability of night shelters for the shelterless population
	• Work with the Department of Education to advocate TB in school health programs and youth awareness clubs
	• Facilitate provision of social protection through available National Health Insurance schemes for below poverty line families and senior citizens. Development of a sustainable program for daily wagers with the Department of Labour
	• Coordinate with the Food and Supplies Department for access to subsidized public distribution system
	• Link with mother and child health services and support networks
	• Establish innovative schemes in public-private partnership
	• Reduce out-of-pocket expenses incurred by people on transport and wage loss by linking with available Social Welfare programs, especially for commuters from satellite towns bordering the city
	• Explore the utilization of existing physical infrastructure for community services
	• Seek opportunities to participate in city development plans and in planning for improvement of medical infrastructure in secondary/tertiary institutes
	• Liaison with the Department of Information and Technology to improve access to digital technology
	• Share best practices with other public health programs to reach out to the vulnerable and marginalized groups in the city
**Stress on affirmative inclusion in TB program**	• ‘Search TB’ in vulnerable and high risk groups among city dwellers
	• Mandatory TB notification by all sectors
	• Support incorporation of basic socio-economic data of patients in TB program surveillance records
	• Develop social inclusion as a separate standard in the International Standards of TB care
	• Incorporate available social welfare schemes in Patient Charter for TB care

## Conclusions

The TB control program worldwide is sincere about addressing the universal health coverage mandate. Therefore, it is essential that program policymakers take cognizance of the fact that in addition to the primacy of public-funded provision and private sector collaboration, explicit strategies for the holistic systemic intervention to health care needs to be drafted by empowering the community first. It is important that strategies which promote social infrastructure development initiatives having a positive impact on TB control, gain momentum to find adequate weightage in the overall policy framework.

### Limitations of the study

Being a metropolitan city with a floating denominator as its population estimates, the number of cases belonging to a specified cohort may not denote the same population. In addition, there are a substantial number of TB cases in the community which are not reported under the program surveillance records. A rough sketch of approximate numbers of such unreported cases could be drawn based on expert opinion; however, to use it for the calculation of decline in disease incidence from the year 2001 would be erroneous and hence not attempted. Nevertheless, it is acknowledged as a limiting factor for further debate.

In the study, ecological analysis has not been attempted; analysis is limited to the program records and published data. Thus, the findings may not represent a true correlation between individual social reform and active TB. Despite these caveats, the synergy between social sector reforms and success of the TB program emerges as a potent force of TB control in urban settings.

## Endnotes

^a^Mission Convergence Program, started by Government of Delhi in the year 2008, is an attempt at holistic planning for social service delivery. With a view to smoothen the process of implementation across various welfare schemes, the Government of Delhi has initiated several steps towards redirecting the governance system so that there is a clear focus on service delivery and enhancement of system efficiency. The most prominent of these steps include a redefinition of the poor into a holistic category of the vulnerable, a targeted vulnerability survey, introduction of electronic beneficiary cards, and a unique public-private partnership at the community level and setting up single window facilitation centres called the Gender Resource Centres – ‘Samajik Suvidha Kendras’. The latter are to work for both empowerment and survey facilitation. This flagship program of the Delhi Government aims to make Delhi a more inclusive city by integrating the existing social security schemes and delivering them through a unified structure in a decentralized manner. More information about this program can be found at http://delhi.gov.in/wps/wcm/connect/DoIT_MC/doit_mc/home.

^b^Delhi Arogya Nidhi Scheme is a State Illness Assistance fund which provides financial assistance to poor patients suffering from life threatening disorders for their treatment in government hospitals (broadly in line with guidelines set by Government of India in the year 1996). By 2011, financial assistance of over INR 200 million has been given to seriously ill patients belonging to below the poverty line in Delhi. More information about the scheme can be found at http://www.delhi.gov.in/wps/wcm/connect/doit_health/Health/Home/Delhi+Arogya+Nidhi.

^c^Laadli Scheme is a demand-based intervention for incentivizing mandatory education of the girl child. The scheme was launched in Delhi in 2008 to empower girls by linking financial assistance with their education up to senior secondary level. Since 2008, 275,651 girls have been registered and have availed the financial benefits of this scheme in Delhi. In the first year of its roll out, the number of female births in Delhi registered per 1,000 boys born increased to 1,004 girl registrations; an increase of 18% from the year 2007. More information about this scheme can be found at http://delhi.gov.in/wps/wcm/connect/doit_wcd/wcd/Home/Delhi+Ladli+Scheme/.

^d^Rashtra Swasthya Bima Yojana is a National health insurance scheme that reduces out-of-pocket expenditure for health care and lessens considerable financial burden on the poorest of the poor. It has helped build the quality chasm in health care delivery as it empowers beneficiaries by providing them with an electronic smart card worth INR 30,000 and empanels hospitals that comply with standard guidelines. More information about this insurance scheme can be found at http://www.delhi.gov.in/wps/wcm/connect/DOIT_Labour/labour/related+links/rashtriya+swasthya+bima+yojana/.

^e^Janani Suraksha Yojana is a conditional cash transfer scheme that incentivizes women to give birth in health facilities. More information about the scheme can be found at http://delhi.gov.in/wps/wcm/connect/doit_health/Health/Home/Family+Welfare/RCH+Programmes/.

^f^Sanjha Prayas is an initiative that was rolled out in 2007 under the Bhagidari program to provide a hygienic atmosphere in Delhi slum clusters. For this purpose, the scheme for providing Financial Assistance to Multi-Purpose Cooperative Societies was rolled out by the Delhi Government. It was envisioned that through this scheme, the Delhi Government would rehabilitate poor people living in slum clusters by providing financial assistance to slum dwellers for their relocation from their existing place or to carry out economic promotional activity at their existing place. More information can be found at http://delhiplanning.nic.in/Write-up/2006-07/V-I/3.pdf.

## Abbreviations

HDI: Human development index; TB: Tuberculosis; WHO: World Health Organization.

## Competing interests

The authors declare that they have no competing interests.

## Authors’ contributions

The study was conceptualized and designed by SC. The original protocol was written by SC and then developed in consultation with NS and KJ. Data collection and analysis was performed by SC supported by NA and NS. Critical review was made by AK, during the several iterations of the manuscript. All authors have approved the final version of the paper. All authors confirm that the manuscript has not been published in any journal or other citable forms.
